# Using an audiovisual feedback device improves cardiopulmonary resuscitation performance during day and night – a randomized controlled simulation study

**DOI:** 10.1186/s12873-025-01249-1

**Published:** 2025-06-07

**Authors:** Melanie Preuss, Rainer Röhrig, Christian Hübel, Clara Vos, Jenny Unterkofler, Jörg Christian Brokmann, Klaus Willmes, Christopher Plata

**Affiliations:** 1 Center for Acute and Emergency Medicine, University Hospital, RWTH Aachen University, Pauwelsstrasse 30, 52074 Aachen, Germany; 2 Institute for Medical Informatics, University Hospital, RWTH Aachen University, Pauwelsstrasse 30, 52074 Aachen, Germany; 3 Department of Neurology, University Hospital, RWTH Aachen University, Pauwelsstrasse 30, 52074 Aachen, Germany

**Keywords:** CPR, BLS, Feedback, T-CPR, In-hospital-cardiac-arrest, Simulation, Manikin, Resuscitation

## Abstract

**Background:**

Survival of in-hospital-cardiac-arrests is lower when they occur at night and at weekends than when they occur during the day. Despite numerous studies, there is little evidence regarding the cardiopulmonary resuscitation quality at night and the influence of a feedback device depending on time of day. The present study investigates the differences between chest compressions at night and during the day, with and without the use of a feedback device.

**Methods:**

The study was approved by the local Ethics Committee and registered in the German Clinical Trials Register on 22nd of February 2022 (DRKS00027309) prior to inclusion of the first participant. 158 medical professionals were randomized into one of two groups: “no feedback” and “feedback”-group. In both groups, participants carried out three two-minute intervals of cardiopulmonary resuscitation on a manikin at day and at night. Members of the “feedback”-group received guidance by a feedback device. Primary endpoint was the mean compression depth at two time-intervals at the beginning (t_1_ = 30–60 s) and the end (t_2_ = 480–540 s) of the experience at night. Secondary endpoints included mean compression depth, adequate compression depth (%), compression rate and effective compressions (%).

**Results:**

At night, mean compression depth was significantly higher in the “feedback”-group at t_1_ (47.7 ± 7.9 mm, 95% CI [45.6–49.8] vs 42.9 ± 11.0 mm, 95% CI [40.8–45.0]) and t_2_ (46.2 ± 7.9 mm, 95% CI [44.0–48.4] vs 39.6 ± 11.6 mm, 95% CI [37.3–41.8]). There was no significant difference in mean compression depth between day and night in the “no feedback”-group (41.4 ± 10.8 mm, 95% CI [39.3–43.5] vs 42.2 ± 10.8 mm, 95% CI [40.1–44.3]) nor in the “feedback-group” (47.4 ± 7.6 mm, 95% CI [45.3–49.4] vs 47.4 ± 7.5 mm; 95% CI [45.4–49.5]).

**Conclusion:**

The use of a real-time audiovisual feedback significantly improved compression depth during the day and night in a manikin-based simulation study with medical professionals.

**Supplementary Information:**

The online version contains supplementary material available at 10.1186/s12873-025-01249-1.

F: 0049 241 33 8,080,804.

## Background

Approximately 290.000 events of in-hospital cardiac arrest (IHCA) occur in the United States every year. Approximately 25% of the patients survive until discharge [1]. In Europe, the incidence for IHCA varies between 1.5 and 2.8 per 1.000 hospital admissions [2]. Therefore, survival to hospital discharge and 30-day-survival differs from 14.8 to 34.0%, depending on initial cardiac rhythm, witness status, place of IHCA, duration of resuscitation, age, and sex [3, 6]. Recent studies suggest that survival from IHCA is lower when cardiac arrest occurs during off-hours compared to occurrence during on-hours [7, 8]. Off-hours are mostly defined as weekdays between 11pm to 6:59am and all day on weekends [8]. Therefore, about half of all IHCA occur during off-hours [8]. Although the underlying reasons for this difference in patients’ survival rates are unknown, different factors might be of importance, e.g. presence of fewer and less qualified hospital staff or an increased number of patients-per-nurse ratio [9, 10]. Previous studies imply that the staff’s physical fitness level might be decreased at night and could potentially influence quality of chest compressions while resuscitating [11, 12]. Since high quality chest compressions are a key factor for an effective CPR and for patient survival [13], nocturnal fatigue could contribute to worse outcomes of IHCA at night. During the daytime, audiovisual feedback devices improve CPR quality [14, 18]. While a previous study found no significant differences in chest compression depth before and after a night shift [19], evidence regarding overall chest compression quality during nighttime hours - and the potential impact of feedback devices used at night - remains limited.

In this study, we hypothesized that nocturnal fatigue of hospital staff influences the quality of chest compressions. Specifically, we examine the influence of a feedback device on nocturnal fatigue while performing CPR. To this end, we analyzed differences in mean compression depth between participants performing CPR at night with and without feedback. Furthermore, we assessed whether chest compression quality varies between daytime and nighttime CPR, and between performances with and without the use of a feedback device.

## Methods

### Study design and setting

The study was approved by the local Medical Faculty Ethics Committee of the RWTH University Aachen, Germany (9th of February 2022, EK 480/21) and registered in the German Clinical Trials Register on 22nd of February 2022 (DRKS00027309) [20] prior to inclusion of the first participant. During this two-armed randomized controlled simulation study, 158 participants performed two repetitive single rescuer basic life support (BLS) scenarios with a simulation manikin (AmbuMan^®^ Advanced, Ambu A/S, Ballerup, Denmark), one during the day and one at night. Depending on the study group, participants performed BLS with or without a feedback device (Zoll X-Series, Zoll, Massachusetts, USA), providing audiovisual real-time feedback. A bar located within the screen provided a visual indication of the current compression depth by moving up and down in synchrony with the chest compressions being performed. Additionally, chest recoil was indicated. The verbal feedback provided to the rescuer included prompts such as “push harder”. A constant metronome beat was set at a rate of 110 beats per minute. Reporting was carried out in accordance with the CONSORT reporting guidelines [21].

## Selection of participants

We randomly recruited medical professionals through face-to-face interactions on the campus of the University Hospital RWTH University, Aachen, Germany. Participants had to be physicians, nursing or emergency medical service (EMS) staff members with a minimum age of 18 and a maximum age of 65 years. Exclusion criteria were pregnancy and refusal. To prevent the Hawthorne effect, participants initially received pseudo-subject education indicating that the study focused on evaluating technical aspects of the resuscitation manikin and feedback sensor - specifically, the detection of varying chest stiffness levels and the accuracy of feedback - in order to divert their attention from their own performance to technical issues. Based on this framing, written informed consent was obtained prior to inclusion, after which participants were randomized into one of the two study groups: “no feedback” or “feedback”. A randomization list was generated by MS Excel 2016 (Microsoft Corporation, Washington, USA) using the randomization function (RAND) with subsequent preparation of envelopes, which contained the randomization number for each participant. Participants were enrolled by a different member of the team to avoid selection bias. Half of each study group started their first performance at night, followed by performance during day and vice versa. The assignment of participants was, in turn, based on a Excel-generated randomization list. A minimum time of 8 h between performances was ensured. After finishing both performances, the participants were informed about the real aim of the study and gave second informed written consent for data processing and analysis. Data privacy for all participants was maintained and pseudonymized data was used for further analyses. Personal data was obtained using a standardized questionnaire (see supplements). The experiments were carried out in a protected area at Aachen University Hospital.

## Interventions

Data was gathered between September 2022 and May 2023. The experiment consisted of two CPR sessions, one at night (between 0 am and 6 am) and one during the day (between 6 am and 8 pm). As a scenario, participants would find themselves in a simulated single rescuer emergency situation of in-hospital cardiac arrest. Besides chest compressions, ventilation was performed with a prefixed endotracheal tube and an enclosed ventilation bag during Basic Life Support. Depending on the study group, participants performed two sessions of BLS with or without the feedback device. The manikin was placed on the floor. While participants in the “no feedback”-group did not get any feedback during the experiment, members of the “feedback”-group also received an introduction to the feedback device and were instructed to pay attention to the audiovisual feedback. For participants in the “feedback”-group, the feedback sensor was taped on the manikin’s chest prior to beginning compressions. Audiovisual feedback provided information about the compression depth, compression frequency and the correct chest recoil. Except for instructions about the time left in each cycle, participants performed chest compressions without additional support during the experiment. During each session, subjects of both study groups performed ten minutes of single rescuer BLS. We divided the 10-minute test period into five intervals of two minutes. During the first period, participants performed chest compressions. After two minutes, participants continued with a two-minute period of ventilation, serving as a repose from physical activity to achieve a guideline compliant level of physical strain. Participants alternated between two minutes of chest compression and two minutes of ventilation until three cycles of CPR were achieved and ten minutes had passed. Prior to physical performance, participants filled out a questionnaire asking about parameters possibly influencing CPR quality, such as gender, age, professional experience as well as their profession and body mass index (BMI) (see supplements). For each session of simulated CPR, participants rated their subjective physical fitness level on a scale ranging from minimum 1 (very poor) to maximum 10 (very good).

## Outcomes

Chest compression depth in millimeters was continuously recorded by the simulation manikin with a rate of 40 Hz (i.e. one data point every 25 milliseconds). As a primary outcome parameter, we compared the mean compression depth with and without feedback at night at two time intervals: t_1_ was set at the beginning of the experiment from second 30 to 90 over a period of 60 s. The second interval (t_2_) was set at the end of the experiment, from second 480 to 540. To prevent data loss towards the end of the experiment resulting from premature termination due to physical exhaustion, the t_2_-interval was set at the beginning of the third CPR cycle (see Figure_s_1 in the supplements). Definitions of chest compression parameters are provided in the supplements. As a secondary outcome, we investigated the influence of time of day on the quality of chest compressions. We therefore analyzed the following parameters during the three two-minute cycles of chest compressions during the day and at night respectively: mean compression depth, number of chest compressions, compressions with adequate, non-sufficient and too deep compression depth, chest compressions with correct chest recoil of the chest, chest compressions with correct positioning of the hand on the thorax, mean compression frequency, effective chest compressions and no-flow time. Effective chest compressions are defined as compressions that meet al.l three criteria: correct compression depth, correct hand position, and complete chest recoil of the thorax. The exact definition of parameters can be found in the supplements. The recorded parameters were also analyzed over time throughout the first, second and third chest compression cycle. The data set and software are available in an online repository [22]. Finally, we included the information from a questionnaire on age, gender, body mass index (BMI), profession and subjective physical fitness to investigate their correlation with the compression parameters (see supplements).

### Statistical analysis

Based on a power level of 80% and an adjusted significance level of 5% for compression depth and frequency, a minimum difference of 5 mm in chest compression depth and 5.7 compressions per minute in compression rate between the two study groups was assumed, according to findings from previous studies [23, 24]. By using the Satterthwaite two-tailed t-test, the required sample size was at 81 per group for chest compressions and 78 for compression rate respectively (SAS 9.3, Cary, North Carolina, USA). The participants’ questionnaire responses about possible parameters influencing CPR quality were analyzed descriptively. Thereby, normally distributed data is presented as mean ± standard deviation (SD). Quantitative data not following a normal distribution is presented as median and interquartile range (IQR). We tested differences in variability between study groups using Levene’s test for quality of variances. For the analysis of the primary endpoint, we performed a two-factorial ANOVA (analysis of variance) with one repeated measures factor (time points) and a grouping factor (feedback/no feedback). The intraclass correlation (ICC, two random factors, absolute agreement) between the two time-points was also analyzed separately for the two groups “feedback” and “no feedback” to obtain information on the stability of compression depth. A three-factorial ANOVA with two repeated measurement factors (time points day, night; compression cycles, 1–3) and the grouping factor feedback and median BMI was performed to analyze secondary endpoints. Post-hoc analysis was carried out with Bonferroni-correction. A p-value < 0.05 was considered to indicate a significant deviation from the respective null hypothesis. The secondary endpoint was selected for the linear regression analysis, as not all subject characteristics were applicable where the primary endpoint was concerned. First, a simple linear regression was performed for each parameter, followed by a multiple linear regression analysis. All data were gathered in anonymous form and SPSS (version 29) was used for statistical analyses.

## Results

### Characteristics of study participants

**Fig. 1:**
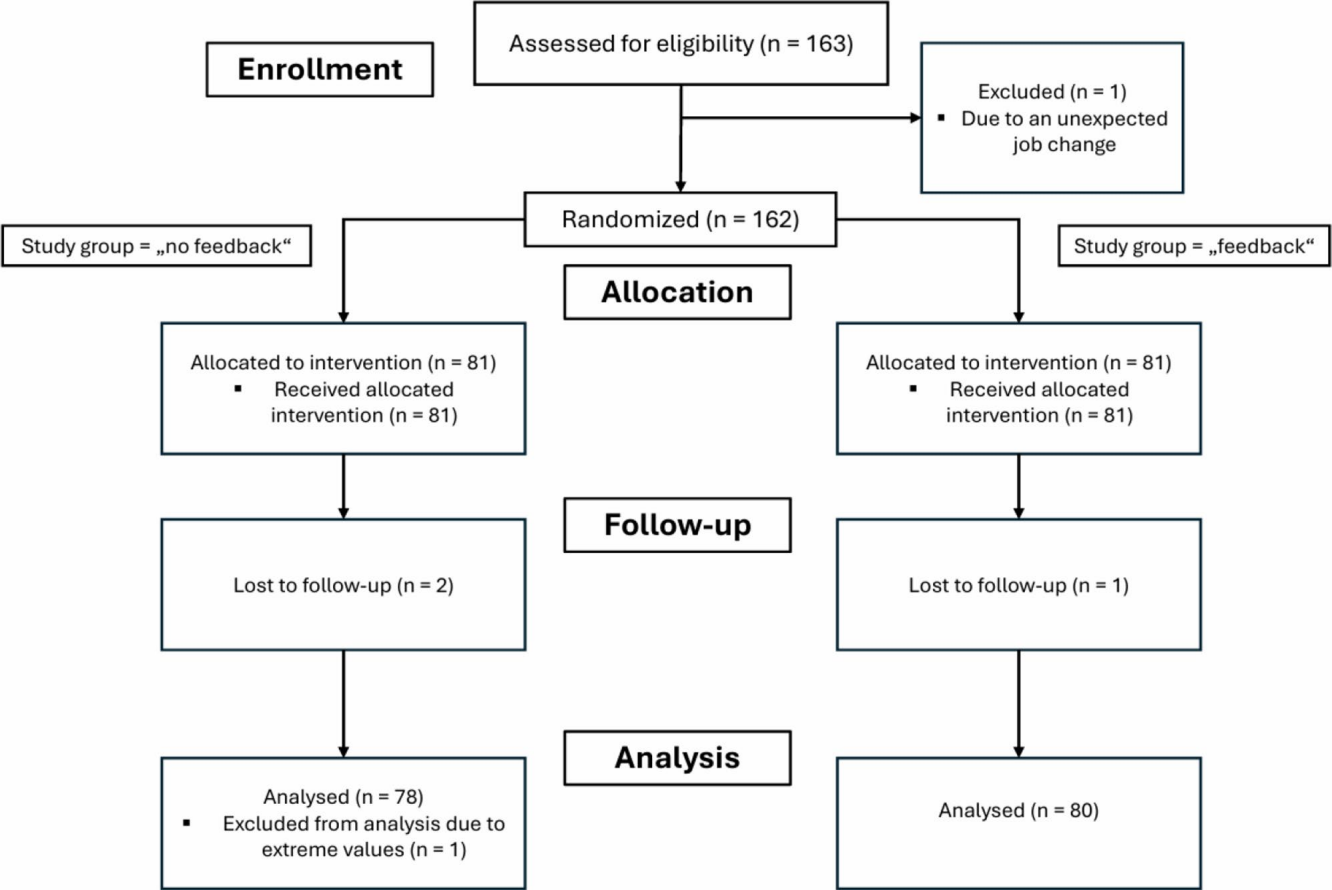
Study diagram

In total, 163 participants were recruited. Participant characteristics can be found in Table 1. Due to an unexpected job change, one participant did not take part in the study despite written consent and randomization and was replaced. Three participants did not appear for the second CPR session due to scheduling difficulties. Because of a lack of written consent after unblinding, these data sets were excluded from statistical analysis. The results of another participant were excluded due to continued insufficient chest compression depth below 20 mm throughout an extended period. Finally, 158 data sets were included and analyzed, 78 in the “no feedback”-group and 80 in the “feedback”-group [Fig. 1].

**Table 1:** Descriptive overview

Characteristic	No feedback (*n* = 78)	Feedback (*n* = 80)
Age (years)	29.0 (26.0–32.0)	29.0 (24.0–34.0)
Gender – n (%) Female Male	34 (44) 44 (56)	33 (41) 47 (59)
Profession – n (%) ^1^ Nursing staff Physicians EMS staff	38 (49) 25 (32) 15 (19)	43 (54) 21 (26) 16 (20)
Professional experience (years)	4.0 (2.0–7.3)	5.0 (2.0–8.4)
BMI (kg/m*)	23.8 (21.2–26.0)	23.8 (21.5–27.8)
Physical Fitness ^2^ Day Night	7.0 (6.0–8.0) 6.0 (5.0–7.0)	8.0 (6.0–8.0) 6.0 (5.0–7.0)

If not otherwise specified data is shown as median (IQR; 25th – 75th percentile) [1] Percentages may not add up 100% due to rounding ^2^ 1 = subjective minimal fitness, 10 = subjective maximal fitness

### Primary endpoint

**Fig. 2:**
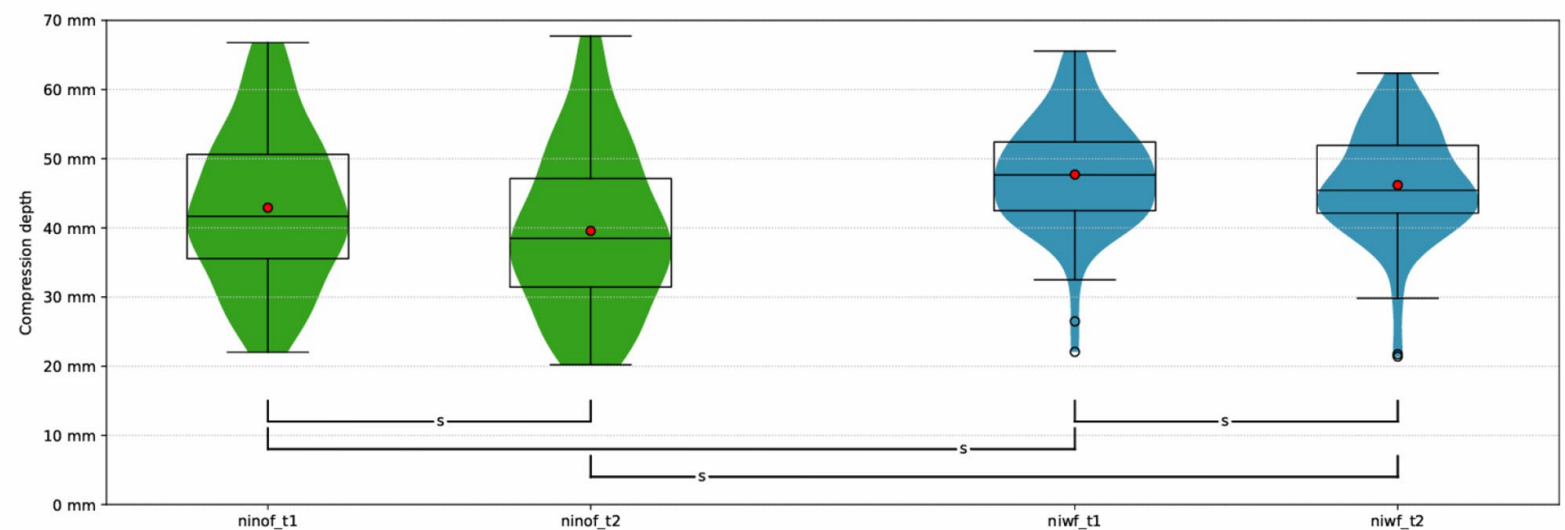
Primary endpoint (mean compression depth at night at t1 and t2). (ninof_t1 = night no feedback first time interval, ninof_t2 = night no feedback second time interval, niwf_t1 = night with feedback first time interval, niwf_t2 = night with feedback second time, s = significant)

There was a significant difference in compression depth between participants performing with feedback and without feedback at t_1_ (47.7 ± 7.9 mm, 95% CI [45.6–49.8] vs. 42.9 ± 11.0 mm, 95% CI [40.8–45.0]) and t_2_ (46.2 ± 7.9 mm, 95% CI [44.0–48.4] vs. 39.6 ± 11.6 mm, 95% CI [37.3–41.8]) (Fig. 2). Mean compression depth in the “no feedback”-group at night decreased significantly between t_1_ and t_2_ (3.4 ± 0.5 mm, 95% CI [2.3–4.4]). In the “feedback”-group, a significant decrease was observed between t_1_ and t_2_ (1.5 mm ± 0.5 mm [0.5–2.5]) (Fig. 2). The intraclass correlation coefficient (ICC) was 0.879 in the “no feedback”-group (95% CI 0.816–0.921) and 0.902 in the “feedback”-group (95% CI 0.851–0.936) (see supplements primary endpoint).

### Secondary endpoints

#### Mean compression depth over time

**Fig. 3:**
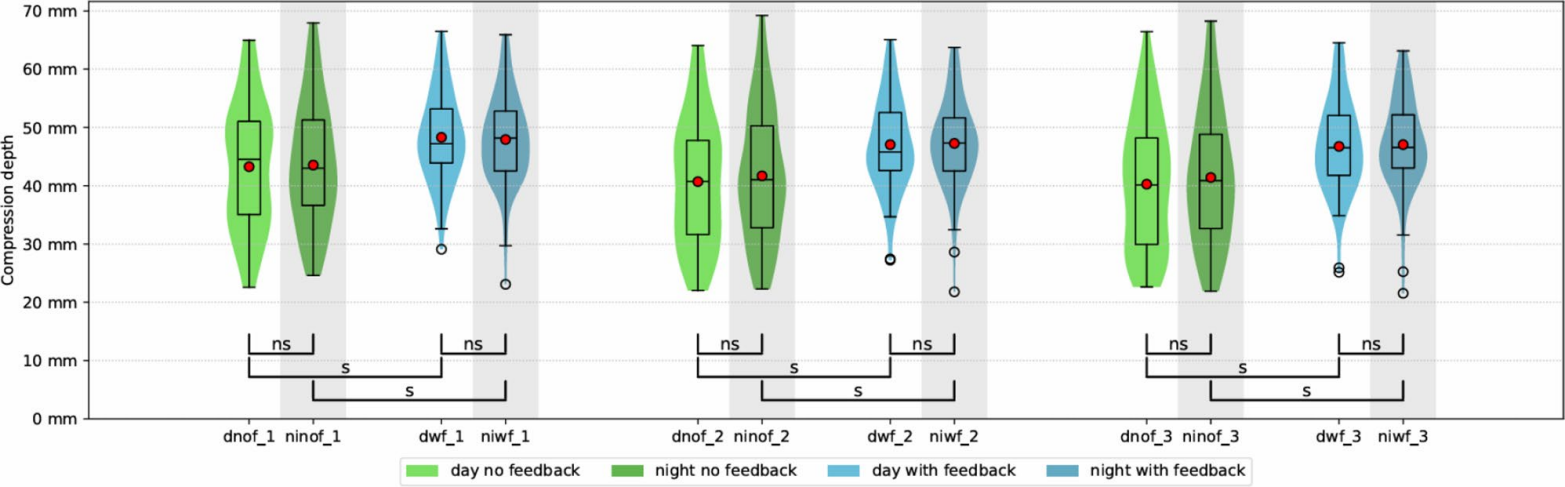
Mean compression depth over time. (dnof_1: day without feedback cycle one, ninof_1: night without feedback cycle one, dwf_1: day with feedback cycle one, niwf_1: night with feedback cycle one, dnof_2: day without feedback cycle two, ninof_2: night without feedback cycle two, dwf_2: day with feedback cycle two, niwf_2: night with feedback cycle two, dnof_3: day without feedback cycle three, ninof_3: night without feedback cycle three, dwf_3: day with feedback cycle three, niwf_3: night with feedback cycle. Grey background indicates CPR at night, ns = not significant, s = significant)

Over the three cycles during the day, mean compression depth in the “feedback”-group differed significantly from the “no feedback”-group (47.4 ± 7.6 mm, 95% CI [45.3–49.4] vs. 41.4 ± 10.8 mm, 95% CI [39.3–43.5]) (see supplements secondary endpoint). At night, a significant difference between the two study groups was also evident (47.4 ± 7.5 mm, 95% CI [45.4–49.5] vs. 42.2 ± 10.8 mm, 95% CI [40.1–44.3]). Comparing compression depths of the “no feedback”-group, there was no significant difference between day and night (41.4 ± 10.8 mm, 95% CI [39.3–43.5] vs. 42.2 ± 10.8 mm 95% CI [40.1–44.3]). Also, within the “feedback”-group, no significant difference in mean compression depth was found between day and night (47.4 ± 7.6 mm 95% CI [45.3–49.4] vs. 47.4 ± 7.5 mm, 95% CI [45.4–49.5]). The “feedback”-group showed a significantly higher mean compression depth in each of the three cycles compared to the “no feedback”-group. This applies to daytime and nighttime (Fig. 3). Further details can be found in the supplements. Participants’ BMI had a significant influence on compression depth, showing a higher mean compression depth if it was greater than 23.8 (41.5 ± 9.0 mm, 95% CI [39.6–43.3] vs. 47.7 ± 8.3 mm, 95% CI [45.9–49.6]).

### Chest compressions with adequate compression depth over time

**Fig. 4:**
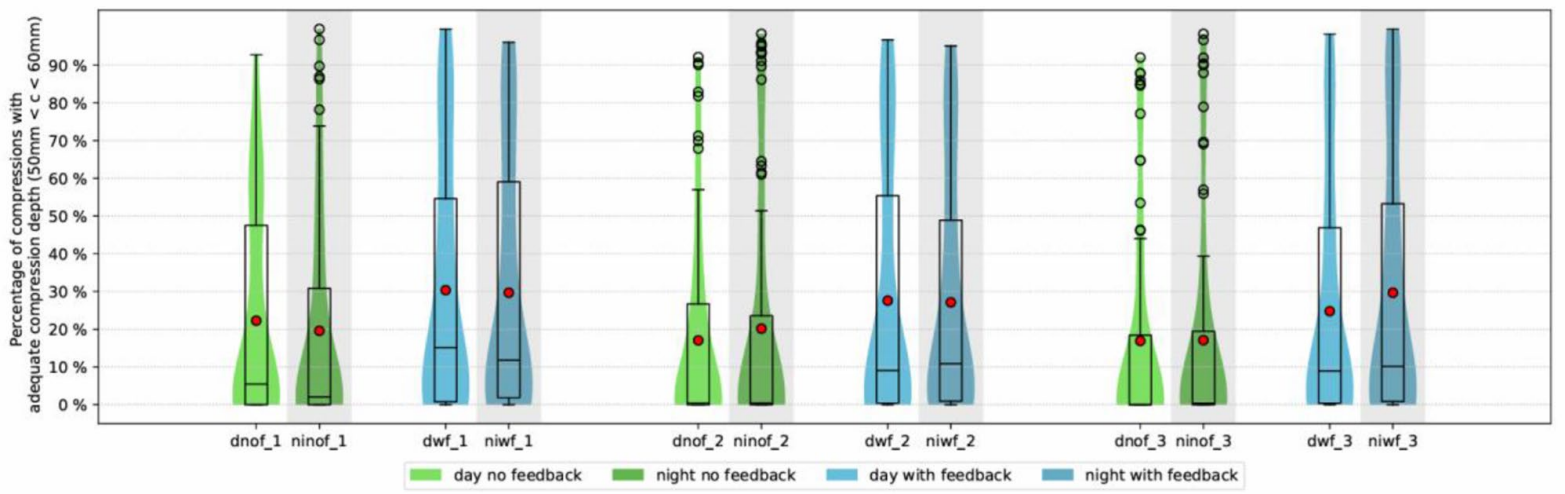
Chest compressions with adequate compression depth over time (%). (dnof_1: day without feedback cycle one, ninof_1: night without feedback cycle one, dwf_1: day with feedback cycle one, niwf_1: night with feedback cycle one, dnof_2: day without feedback cycle two, ninof_2: night without feedback cycle two, dwf_2: day with feedback cycle two, niwf_2: night with feedback cycle two, dnof_3: day without feedback cycle three, ninof_3: night without feedback cycle three, dwf_3: day with feedback cycle three, niwf_3: night with feedback cycle)

There was a significant difference in mean chest compressions with adequate compression depth in relation to the numbers of performed compressions between the “feedback” and “no feedback”-group (63.3 ± 64.4, 95% CI [50.2–76.5] (28.2%) vs 42.9 ± 54.0, 95% CI [29.5–56.2] (18.8%)). In the “no feedback”-group, no significant difference was found between day and night (42.8 ± 59.0, 95% CI [28.1–57.6] (18.7%) vs 42.9 ± 65.5, 95% CI [27.9–57.9] (19.0%)). No such difference was found in the “feedback”-group either (62.3 ± 71.9, 95% CI [47.7–76.8] (27.6%) vs. 64.4 ± 68.6, 95% CI [49.5–79.2](28.8%)) [Fig. 4].

### Compression rate

**Fig. 5:**
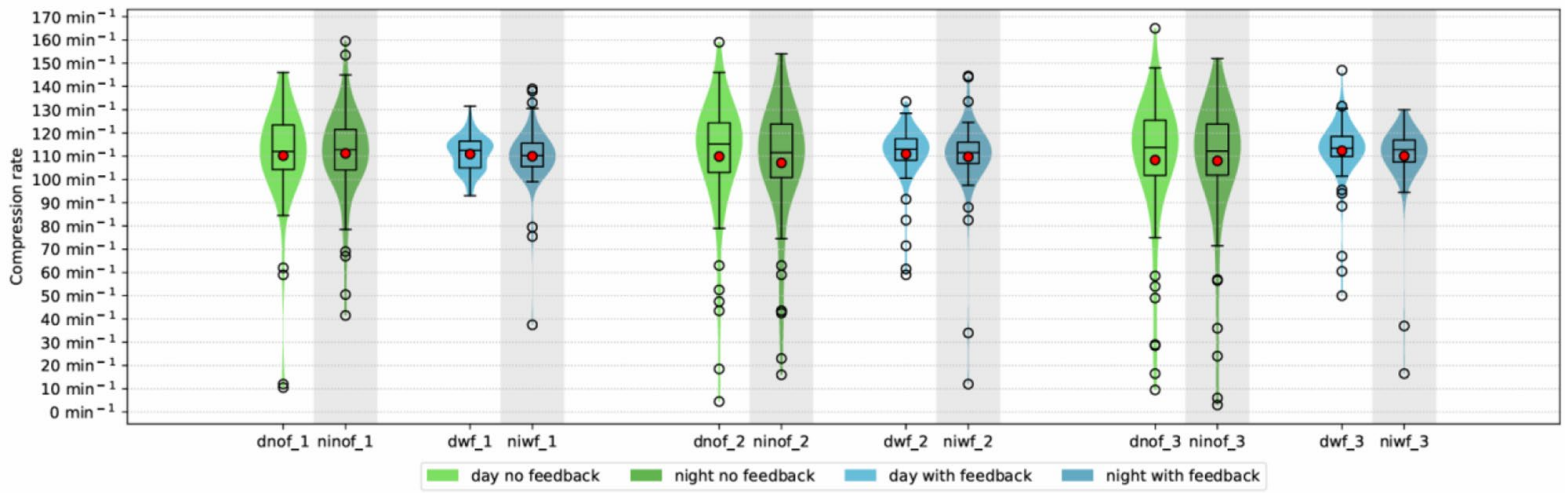
Compression rate. (dnof_1: day without feedback cycle one, ninof_1: night without feedback cycle one, dwf_1: day with feedback cycle one, niwf_1: night with feedback cycle one, dnof_2: day without feedback cycle two, ninof_2: night without feedback cycle two, dwf_2: day with feedback cycle two, niwf_2: night with feedback cycle two, dnof_3: day without feedback cycle three, ninof_3: night without feedback cycle three, dwf_3: day with feedback cycle three, niwf_3: night with feedback cycle)

Over the three cycles of chest compressions, there was no significant difference in compression rate between “feedback” and “no feedback”-group (110.7 ± 11.2, 95% CI [107.0–114.4] vs. 109.1 ± 21.1, 95% CI [105.4–112.9]). Mean compression frequency in both study groups was within the recommended range of 100 to 120 per minute [Fig. 5].

### Effective compressions

**Fig. 6:**
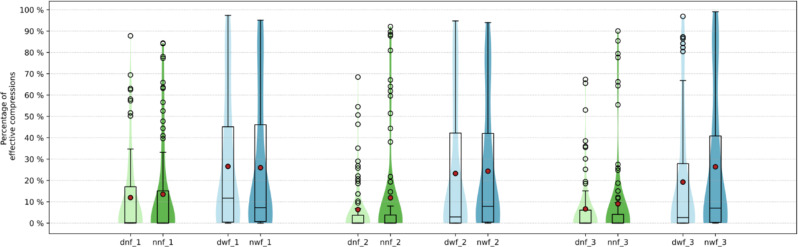
Effective compressions. ((%) as composite value of compressions with correct depth of pressure, correct hand position and subsequent correct chest recoil of the chest, dnof_1: day without feedback cycle one, ninof_1: night without feedback cycle one, dwf_1: day with feedback cycle one, niwf_1: night with feedback cycle one, dnof_2: day without feedback cycle two, ninof_2: night without feedback cycle two, dwf_2: day with feedback cycle two, niwf_2: night with feedback cycle two, dnof_3: day without feedback cycle three, ninof_3: night without feedback cycle three, dwf_3: day with feedback cycle three, niwf_3: night with feedback cycle)

A significant difference in effective compressions was found between the “feedback” and “no feedback”-group (62.9 ± 64.4, 95% CI [50.0–75 − 8] (24.3%) vs. 36.9 ± 51.2, 95% CI [23.8–49.9] (9.9%)) [Fig. 6].

### Ancillary analyses

We did not find significant differences between the “feedback” and “no feedback”-group regarding number of compressions with non-sufficient compression depth (141.1 ± 80.3, 95% CI [123.6–158.6] vs. 160.0 ± 77.8, 95% CI [142-3–177.6]), number of compressions with too deep compression depth (16.9 ± 44.4, 95% CI [7.0–26.9] vs. 15.4 ± 45.5, 95% CI [5.4–25.5]) and number of compressions with correct chest recoil (220.2 ± 22.5, 95% CI [212.6–227.8] vs. 215.9 ± 43.5[208.2–223.6]). Significant differences were found regarding the number of chest compressions with correct hand position (220.6 ± 22.1, 95% CI [210.4–230.8] vs. 196.1 ± 61.9, 95% CI [185.8–206.5]) and cumulative “no-flow”-time (2.6 ± 9.1, 95% CI [0.014 -5.3] vs. 6.9 ± 14.2, 95% CI [4.3–9.6]). After conducting a linear regression analysis, we found a significant influence of median BMI (> 23.8), gender and subjective physical fitness on mean compression depth during the day and night. The influence of BMI and gender remained significant after performing a multiple linear regression analysis. Further details can be found in the supplements.

## Discussion

The results of the present study show that the use of a feedback device at night significantly increased the mean compression depth at the beginning (t_1_) and towards the end of the ten-minute experiment (t_2_) in comparison with the “no feedback”-group. A significant decrease in the mean compression depth between t_1_ and t_2_ was found within the “feedback” and the “no feedback” group. Over the three cycles, chest compression depth was significantly higher in all three cycles in the “feedback”-group during the day and at night compared to the “no feedback”-group. No significant differences in mean compression depth were found between day and night.

We found signs of physical exhaustion while performing CPR at night with and without the use of a feedback device. However, the decrease in mean compression depth in the “no feedback”-group was twice the decrease in the “feedback”-group (3.4 mm vs. 1.5 mm). Similar results were found in a simulation study with health care professionals performing CPR on a manikin: while participants performing with a feedback device did not show significant differences between minute 1 and 10, participants performing without the feedback device showed a decreased quality of chest compression depth over time [14]. It should be noted that the isolated use of a feedback device is not recommended and should be combined with other measures, such as quality improvement programs [25].

Surprisingly, mean compression depth, with or without the use of a feedback device, did not reach the recommended minimum of 50 mm by the ERC [13]. Naturally, not reaching the recommended compression depth raises a concern about the quality of chest compressions provided by healthcare professionals and should be addressed with team training and simulation and simulation practice. But our trial design may also provide an explanation for this phenomenon. To emphasize the effect of physical exhaustion and to show the maximum effect of fatigue and feedback effect respectively, the resistance of the manikin’s chest was set to maximum rigidity. However, this level of resistance can be found in real patients, e.g. young, muscular or obese patients.

Surprisingly, the mean compression depth - regardless of whether a feedback device was used - did not reach the minimum of 50 mm recommended by the ERC guidelines [13]. This shortfall raises legitimate concerns regarding the quality of chest compressions delivered by healthcare professionals and underscores the need for targeted team training and simulation-based practice. However, our study design may partially account for this finding. In order to emphasize the impact of physical exhaustion and to isolate the effects of fatigue and real-time feedback, the manikin’s chest stiffness was set to its maximum resistance. Notably, such resistance levels are not purely theoretical but may be encountered in clinical practice, particularly in young, muscular, or obese patients. An additional noteworthy observation concerning the interaction between the feedback sensor and the simulation manikin emerged prior to the commencement of the experiment. Thereby, we found remarkable differences between the compression depth that was indicated by the feedback monitor and the feedback surface of the manikin on a laptop. This difference was smallest and tolerable, when the highest level of resistance was chosen for the manikin’s chest. A possible explanation could be found in the accelerometer of the feedback sensor and its fundamental algorithms, which are adjusted to human chest characteristics. Hence, false compression levels might be found due to the interaction of the feedback sensor and the artificial chest of the simulator. Therefore, we must conclude that our results of mean compression depth are systematically lower. The question can be raised if other authors might have had the same challenge but did not detect the issue.

We did not observe significant differences in CPR performance between day and night sessions. Our findings do not support the hypothesis that reduced CPR quality during nighttime hours is caused by nocturnal fatigue. However, it is important to emphasize that CPR quality represents only one of multiple factors influencing survival after IHCA. Moreover, as this was a simulation-based study, our findings cannot be directly extrapolated to patient outcomes such as survival or neurological recovery. Rather, they provide controlled experimental evidence regarding isolated aspects of resuscitation performance under standardized conditions. Recent exploration found possible causes in delayed calls of emergency teams at night [26] and increased number of patients-per-nurse at night [9, 10]. For this study, mean compression depth was selected as the primary outcome parameter. It may be argued, however, that composite parameters such as “effective compressions” or “chest compression fraction,” which encompass multiple dimensions of CPR quality, could better reflect clinical relevance. In future studies, such parameters may provide a more meaningful assessment of resuscitation performance than isolated metric values alone.

### Limitations

The calculated sample size for assessing chest compression depth was not fully achieved. Nevertheless, we observed a statistically significant effect of the feedback device. While this represents a limitation, it may be reasonably argued that the inclusion of four additional participants would be unlikely to meaningfully alter the observed effect or change the overall conclusions of the study. Measurements took place in a laboratory setting and thus do not represent real-life conditions. Due to the study design, participants were not surprised by an emergency. Thus, potential confounders like preparedness and lower individual stress level may have influenced the results. Possibly, participants did not find themselves at their physiological low point regarding physical performance because most of the participants performed nocturnal CPR between 0am and 2am.

## Conclusion

In summary, the use of a real-time feedback device in a simulation manikin study increases chest compression depth during the day and at night.

## Electronic supplementary material

Below is the link to the electronic supplementary material.

Supplementary Material 1

Supplementary Material 2

Supplementary Material 3

Supplementary Material 4

Supplementary Material 5

## Data Availability

The datasets generated and analyzed during the current study are available in the RWTH publications repository 10.18154/RWTH-2024-08349.

## Abbreviations

CPR: Cardiopulmonary resuscitation
IHCA: In-hospital-cardiac arrest
BLS: Basic life support
CONSORT: Consolidated Standards of Reporting Trails
EMS: Emergency medical services
BMI: Body mass index
SD: Standard deviation
IQR: Interquartile range
ANOVA: Analysis of variance

## References

[1] Andersen LW, Holmberg MJ, Berg KM, Donnino MW, Granfeldt A. In-Hospital cardiac arrest: A review. JAMA - J Am Med Association. 2019;321(12):1200–10. 10.1001/jama.2019.1696. 10.1001/jama.2019.1696 PMC6482460 30912843

[2] Perkins GD, Graesner JT, Semeraro F, et al. European resuscitation Council guidelines 2021: executive summary. Resuscitation. 2021;161:1–60. 10.1016/j.resuscitation.2021.02.003. 33773824 10.1016/j.resuscitation.2021.02.003

[3] Widestedt H, Giesecke J, Karlsson P, Jakobsson JG. In-hospital cardiac arrest resuscitation performed by the hospital emergency team: A 6-year retrospective register analysis at danderyd university hospital, Sweden. F1000Res. 2018;7:1013. 10.12688/f1000research.15373.1. 30356455 10.12688/f1000research.15373.1 PMC6178903

[4] Piscator E, Hedberg P, Göransson K, Djärv T. Survival after in-hospital cardiac arrest is highly associated with the Age-combined Charlson Co-morbidity index in a cohort study from a two-site Swedish university hospital. Resuscitation. 2016;99:79–83. 10.1016/j.resuscitation.2015.11.023. 26708451 10.1016/j.resuscitation.2015.11.023

[5] Nolan JP, Soar J, Smith GB, et al. Incidence and outcome of in-hospital cardiac arrest in the united Kingdom National cardiac arrest audit. Resuscitation. 2014;85(8):987–92. 10.1016/j.resuscitation.2014.04.002. 24746785 10.1016/j.resuscitation.2014.04.002

[6] Radeschi G, Mina A, Berta G, et al. Incidence and outcome of in-hospital cardiac arrest in Italy: a multicentre observational study in the Piedmont region. Resuscitation. 2017;119:48–55. 10.1016/j.resuscitation.2017.06.020. 28655621 10.1016/j.resuscitation.2017.06.020

[7] Peberdy MA. Survival from In-Hospital cardiac arrest during nights and weekends. JAMA. 2008;299(7):785. 10.1001/jama.299.7.785. 18285590 10.1001/jama.299.7.785

[8] Ofoma UR, Basnet S, Berger A, et al. Trends in survival after In-Hospital cardiac arrest during nights and weekends. J Am Coll Cardiol. 2018;71(4):402–11. 10.1016/j.jacc.2017.11.043. 29389356 10.1016/j.jacc.2017.11.043 PMC5858924

[9] Hegarty H, Knight T, Atkin C, et al. Nurse staffing levels within acute care: results of a National day of care survey. BMC Health Serv Res. 2022;22(1):493. 10.1186/s12913-022-07562-w. 35418056 10.1186/s12913-022-07562-w PMC9008904

[10] Rosa-Zamboni D, de la, Carrasco-González MI, de Blas-Barrientos N, et al. Patient-nurse ratio as an index related to healthcare-associated infections: a surveillance study. Bol Med Hosp Infant Mex. 2023;80(1):29–35. 10.24875/BMHIM.22000117. 36867569 10.24875/BMHIM.22000117

[11] Smith-Coggins R, Rosekind MR, Buccino KR, Dinges DF, Moser RP. Rotating shiftwork schedules: can we enhance physician adaptation to night shifts? Acad Emerg Med. 1997;4(10):951–61. 10.1111/j.1553-2712.1997.tb03658.x. 9332626 10.1111/j.1553-2712.1997.tb03658.x

[12] Barger LK, Ayas NT, Cade BE, et al. Impact of extended-duration shifts on medical errors, adverse events, and attentional failures. PLoS Med. 2006;3(12):e487. 10.1371/journal.pmed.0030487. 17194188 10.1371/journal.pmed.0030487 PMC1705824

[13] Olasveengen TM, Semeraro F, Ristagno G, et al. European resuscitation Council guidelines 2021: basic life support. Resuscitation. 2021;161:98–114. 10.1016/j.resuscitation.2021.02.009. 33773835 10.1016/j.resuscitation.2021.02.009

[14] Buléon C, Delaunay J, Parienti JJ, et al. Impact of a feedback device on chest compression quality during extended manikin CPR: a randomized crossover study. Am J Emerg Med. 2016;34(9):1754–60. 10.1016/j.ajem.2016.05.077. 27349359 10.1016/j.ajem.2016.05.077

[15] Buléon C, Parienti JJ, Halbout L, et al. Improvement in chest compression quality using a feedback device (CPRmeter): a simulation randomized crossover study. Am J Emerg Med. 2013;31(10):1457–61. 10.1016/j.ajem.2013.07.029. 24035507 10.1016/j.ajem.2013.07.029

[16] Lee PH, Lai HY, Hsieh TC, Wu WR. Using real-time device-based visual feedback in CPR recertification programs: A prospective randomised controlled study. Nurse Educ Today. 2023;124:105755. 10.1016/j.nedt.2023.105755. 36863107 10.1016/j.nedt.2023.105755

[17] Baldi E, Cornara S, Contri E, et al. Real-time visual feedback during training improves laypersons’ CPR quality: a randomized controlled manikin study. CJEM. 2017;19(6):480–7. 10.1017/cem.2016.410. 28115027 10.1017/cem.2016.410

[18] Wee JCP, Nandakumar M, Chan YH, et al. Effect of using an audiovisual CPR feedback device on chest compression rate and depth. Ann Acad Med Singap. 2014;43(1):33–8. 24557463

[19] Philippon AL, Nguyen A, Raynal PA, et al. Weaker compressions after night shift? The WeCAN manikin study. Eur J Emerg Med. 2016;23(1):65–7. 10.1097/MEJ.0000000000000284. 25969346 10.1097/MEJ.0000000000000284

[20] Trial registration in the German Clinical Trials Register. Accessed October 3. 2024. https://drks.de/search/en/trial/DRKS00027309

[21] Schulz KF, Altman DG, Moher D, Group CONSORT. CONSORT 2010 statement: updated guidelines for reporting parallel group randomised trials. BMJ. 2010;340:c332. 10.1136/bmj.c332. 20332509 10.1136/bmj.c332 PMC2844940

[22] Online Repository. Accessed October 3. 2024. 10.18154/RWTH-2024-08349

[23] Augusto JB, Santos MB, Faria D, et al. Real-Time visual feedback device improves quality of chest compressions: A manikin study. Bull Emerg Trauma. 2020;8(3):135–41. 10.30476/BEAT.2020.83080. 32944572 10.30476/BEAT.2020.83080 PMC7468227

[24] Vadeboncoeur T, Stolz U, Panchal A, Silver A, Venuti M, Tobin J, Smith G, Nunez M, Karamooz M, Spaite D, Bobrow B. Chest compression depth and survival in out-of-hospital cardiac arrest. Resuscitation. 2014;85(2):182-8. doi: 10.1016/j.resuscitation.2013.10.002. Epub 2013 Oct 12. PMID: 24125742. 10.1016/j.resuscitation.2013.10.002 24125742

[25] Olasveengen TM, Mancini ME, Perkins GD, et al. Adult basic life support: 2020 international consensus on cardiopulmonary resuscitation and emergency cardiovascular care science with treatment recommendations. Circulation. 2020;142(16suppl1):S41–91. 10.1161/CIR.0000000000000892. 33084391 10.1161/CIR.0000000000000892

[26] Chen J, Bellomo R, Flabouris A, Hillman K, Assareh H, Ou L. Delayed emergency team calls and associated hospital mortality: A multicenter study. Crit Care Med. 2015;43(10):2059–65. 10.1097/CCM.0000000000001192. 26181217 10.1097/CCM.0000000000001192

