# Supplementary material for: Using an audiovisual feedback device improves cardiopulmonary resuscitation performance during day and night – a randomized controlled simulation study
Source: BMC Emerg Med. 2025 Jun 7;25:95. doi: 10.1186/s12873-025-01249-1 (PMC12145583; doi:10.1186/s12873-025-01249-1)
Supplement: Supplementary file 1 — Supplementary Material 1 [file 12873_2025_1249_MOESM1_ESM.docx]

**# Supplements: definitions of chest compressions**

- Chest compressions: pressure on the manikin’s chest with an impression depth > 20 mm, starting from the previous minimum until the following maximum
- Minimum: lowest pressure depth within one compression
- Maximum: highest pressure depth within one compression
- Compression depth: maximum pressure amplitude within one compression
- Correct chest recoil of the thorax: minimal pressure depth < 10 mm from the zero line
- Correct hand position: defined and detected by the manikin
- Correct compression frequency: between 100 and 120 compressions per minute
- Non-sufficient compression depth: compressions < 50mm of depth
- Too deep compression depth: = > 60 mm
- Adequate compression depth: between 50 and 60 mm.
- Effective chest compressions: chest compressions with correct compression depth, correct hand position and correct chest recoil of the thorax
- No-flow time: cumulative time when chest compressions were absent, starting after 2 seconds of interruption or if a compression depth of < 20mm was performed for more than 2 seconds, measured from last maximum to next maximum between two valid chest compressions
