# Supplementary figures and images for: Using an audiovisual feedback device improves cardiopulmonary resuscitation performance during day and night – a randomized controlled simulation study
Source: BMC Emerg Med. 2025 Jun 7;25:95. doi: 10.1186/s12873-025-01249-1 (PMC12145583; doi:10.1186/s12873-025-01249-1)

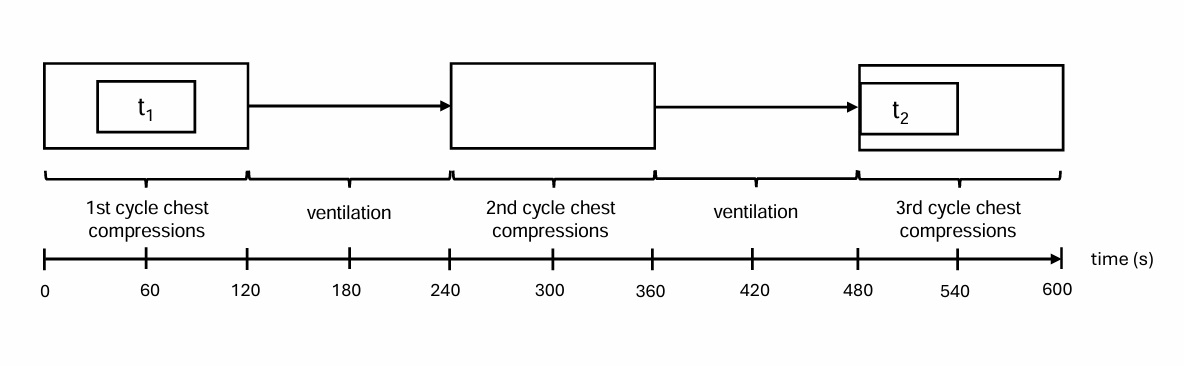

Supplement: Supplementary file 2 — Supplementary Material 2 [file 12873_2025_1249_MOESM2_ESM.jpg]
