# Supplementary material for: Using an audiovisual feedback device improves cardiopulmonary resuscitation performance during day and night – a randomized controlled simulation study
Source: BMC Emerg Med. 2025 Jun 7;25:95. doi: 10.1186/s12873-025-01249-1 (PMC12145583; doi:10.1186/s12873-025-01249-1)
Supplement: Supplementary file 3 — Supplementary Material 3 [file 12873_2025_1249_MOESM3_ESM.docx]

Questionnaire for participating in research project

*Detection of quality differences in resuscitation on a simulation manikin with varying chest mechanics resistance, comparing day and night conditions*

Dear participant,

as part of your participation in the above-mentioned study, we ask you to complete the following questionnaire. Thank you!

Randomization code: _________________

1. Which gender do you identify with?

Male Female Diverse

1. How old are you? _________ years
2. Which healthcare profession group do you belong to?

Nursing staff EMS staff Physician

1. How many years of professional experience do you have? _________years
2. Height ________ cm weight ________ kg
3. How do you currently rate your physical fitness?

(1: very poor; 10: very good)

- 1. At day (______________­­­­__): 1 – 2 – 3 – 4 – 5 – 6 – 7 – 8 – 9 – 10
  2. At night (______________­­­­__): 1 – 2 – 3 – 4 – 5 – 6 – 7 – 8 – 9 – 10

Date and Time (filled in by study staff):
