# Supplementary material for: Using an audiovisual feedback device improves cardiopulmonary resuscitation performance during day and night – a randomized controlled simulation study
Source: BMC Emerg Med. 2025 Jun 7;25:95. doi: 10.1186/s12873-025-01249-1 (PMC12145583; doi:10.1186/s12873-025-01249-1)
Supplement: Supplementary file 4 — Supplementary Material 4 [file 12873_2025_1249_MOESM4_ESM.docx]

**Supplements primary endpoint**

| **Intraclass Correlation Coefficient** | | | | | | | | |
| --- | --- | --- | --- | --- | --- | --- | --- | --- |
| study goup | | Intraclass Correlation^b^ | 95% Confidence Interval | | F Test with True Value 0 | | | |
|  |  |  | Lower Bound | Upper Bound | Value | df1 | df2 | Sig |
| no feedback | Single Measures | .879^a^ | .816 | .921 | 15.493 | 77 | 77 | <.001 |
|  | Average Measures | .935^c^ | .899 | .959 | 15.493 | 77 | 77 | <.001 |
| feedback | Single Measures | .902^a^ | .851 | .936 | 19.433 | 79 | 79 | <.001 |
|  | Average Measures | .949^c^ | .920 | .967 | 19.433 | 79 | 79 | <.001 |
| Two-way mixed effects model where people effects are random and measures effects are fixed. | | | | | | | | |
| a. The estimator is the same, whether the interaction effect is present or not. | | | | | | | | |
| b. Type C intraclass correlation coefficients using a consistency definition. The between-measure variance is excluded from the denominator variance. | | | | | | | | |
| c. This estimate is computed assuming the interaction effect is absent, because it is not estimable otherwise. | | | | | | | | |
