# Supplementary material for: Using an audiovisual feedback device improves cardiopulmonary resuscitation performance during day and night – a randomized controlled simulation study
Source: BMC Emerg Med. 2025 Jun 7;25:95. doi: 10.1186/s12873-025-01249-1 (PMC12145583; doi:10.1186/s12873-025-01249-1)
Supplement: Supplementary file 5 — Supplementary Material 5 [file 12873_2025_1249_MOESM5_ESM.docx]

Supplements secondary endpoint

**Mean compression depth**

| study goup | day_night | Mean | Std. Error | 95% Confidence Interval | |
| --- | --- | --- | --- | --- | --- |
|  |  |  |  | Lower Bound | Upper Bound |
| no feedback | 1 | 41.410 | 1.054 | 39.328 | 43.493 |
|  | 2 | 42.213 | 1.054 | 40.131 | 44.295 |
| feedback | 1 | 47.364 | 1.041 | 45.307 | 49.420 |
|  | 2 | 47.414 | 1.041 | 45.358 | 49.470 |

| Pairwise Comparisons | | | | | | | |
| --- | --- | --- | --- | --- | --- | --- | --- |
| day_night | (I) study goup | (J) study goup | Mean Difference (I-J) | Std. Error | Sig.^b^ | 95% Confidence Interval for Difference^b^ | |
|  |  |  |  |  |  | Lower Bound | Upper Bound |
| 1 | no feedback | feedback | -5.953^*^ | 1.482 | <.001 | -8.880 | -3.026 |
|  | feedback | no feedback | 5.953^*^ | 1.482 | <.001 | 3.026 | 8.880 |
| 2 | no feedback | feedback | -5.201^*^ | 1.481 | <.001 | -8.127 | -2.274 |
|  | feedback | no feedback | 5.201^*^ | 1.481 | <.001 | 2.274 | 8.127 |
| Based on estimated marginal means | | | | | | | |
| *. The mean difference is significant at the .05 level. | | | | | | | |
| b. Adjustment for multiple comparisons: Bonferroni. | | | | | | | |

| Pairwise Comparisons | | | | | | | |
| --- | --- | --- | --- | --- | --- | --- | --- |
| study goup | (I) day_night | (J) day_night | Mean Difference (I-J) | Std. Error | Sig.^a^ | 95% Confidence Interval for Difference^a^ | |
|  |  |  |  |  |  | Lower Bound | Upper Bound |
| no feedback | 1 | 2 | -.803 | .692 | .248 | -2.170 | .565 |
|  | 2 | 1 | .803 | .692 | .248 | -.565 | 2.170 |
| feedback | 1 | 2 | -.050 | .683 | .942 | -1.400 | 1.300 |
|  | 2 | 1 | .050 | .683 | .942 | -1.300 | 1.400 |
| Based on estimated marginal means | | | | | | | |
| a. Adjustment for multiple comparisons: Bonferroni. | | | | | | | |

| Pairwise Comparisons | | | | | | | | |
| --- | --- | --- | --- | --- | --- | --- | --- | --- |
| day_night | cycle | (I) study goup | (J) study goup | Mean Difference (I-J) | Std. Error | Sig.^b^ | 95% Confidence Interval for Difference^b^ | |
|  |  |  |  |  |  |  | Lower Bound | Upper Bound |
| 1 | 1 | no feedback | feedback | -5.038^*^ | 1.492 | <.001 | -7.985 | -2.092 |
|  |  | feedback | no feedback | 5.038^*^ | 1.492 | <.001 | 2.092 | 7.985 |
|  | 2 | no feedback | feedback | -6.344^*^ | 1.508 | <.001 | -9.323 | -3.365 |
|  |  | feedback | no feedback | 6.344^*^ | 1.508 | <.001 | 3.365 | 9.323 |
|  | 3 | no feedback | feedback | -6.477^*^ | 1.542 | <.001 | -9.523 | -3.432 |
|  |  | feedback | no feedback | 6.477^*^ | 1.542 | <.001 | 3.432 | 9.523 |
| 2 | 1 | no feedback | feedback | -4.381^*^ | 1.473 | .003 | -7.290 | -1.472 |
|  |  | feedback | no feedback | 4.381^*^ | 1.473 | .003 | 1.472 | 7.290 |
|  | 2 | no feedback | feedback | -5.577^*^ | 1.527 | <.001 | -8.593 | -2.561 |
|  |  | feedback | no feedback | 5.577^*^ | 1.527 | <.001 | 2.561 | 8.593 |
|  | 3 | no feedback | feedback | -5.644^*^ | 1.543 | <.001 | -8.692 | -2.596 |
|  |  | feedback | no feedback | 5.644^*^ | 1.543 | <.001 | 2.596 | 8.692 |
| Based on estimated marginal means | | | | | | | | |
| *. The mean difference is significant at the .05 level. | | | | | | | | |
| b. Adjustment for multiple comparisons: Bonferroni. | | | | | | | | |

| **Influence of BMI on mean compression depth** | | | | |
| --- | --- | --- | --- | --- |
| BMI_Median | Mean | Std. Error | 95% Confidence Interval | |
|  |  |  | Lower Bound | Upper Bound |
| BMI < 23.816 | 41.481 | .930 | 39.643 | 43.319 |
| BMI >= 23.816 | 47.719 | .930 | 45.881 | 49.557 |

| Pairwise Comparisons | | | | | | |
| --- | --- | --- | --- | --- | --- | --- |
| (I) BMI_Median | (J) BMI_Median | Mean Difference (I-J) | Std. Error | Sig.^b^ | 95% Confidence Interval for Difference^b^ | |
|  |  |  |  |  | Lower Bound | Upper Bound |
| BMI < 23.816 | BMI >= 23.816 | -6.238^*^ | 1.316 | <.001 | -8.837 | -3.638 |
| BMI >= 23.816 | BMI < 23.816 | 6.238^*^ | 1.316 | <.001 | 3.638 | 8.837 |
| Based on estimated marginal means | | | | | | |
| *. The mean difference is significant at the .05 level. | | | | | | |
| b. Adjustment for multiple comparisons: Bonferroni. | | | | | | |

**Ancillary Analysis**

**Multiple linear regression analysis of participants questionnaire**

Dependent variable: mean compression depth at day

| Variables Entered/Removed^a^ | | | |
| --- | --- | --- | --- |
| Model | Variables Entered | Variables Removed | Method |
| 1 | subjective physical fitness day, BMI, age in years, gender, work experience in years^b^ | . | Enter |
| a. Dependent Variable: mean pressure depth in mm 1st + 2nd + 3rd cycle day | | | |
| b. All requested variables entered. | | | |

| Model Summary | | | | |
| --- | --- | --- | --- | --- |
| Model | R | R Square | Adjusted R Square | Std. Error of the Estimate |
| 1 | .560^a^ | .314 | .291 | 8.21039 |
| a. Predictors: (Constant), subjective physical fitness day, BMI, age in years, gender, work experience in years | | | | |

| ANOVA^a^ | | | | | | |
| --- | --- | --- | --- | --- | --- | --- |
| Model | | Sum of Squares | df | Mean Square | F | Sig. |
| 1 | Regression | 4681.524 | 5 | 936.305 | 13.890 | <.001^b^ |
|  | Residual | 10246.403 | 152 | 67.411 |  |  |
|  | Total | 14927.927 | 157 |  |  |  |
| a. Dependent Variable: mean pressure depth in mm 1st + 2nd + 3rd cycle day | | | | | | |
| b. Predictors: (Constant), subjective physical fitness day, BMI, age in years, gender, work experience in years | | | | | | |

| Coefficients^a^ | | | | | | | | |
| --- | --- | --- | --- | --- | --- | --- | --- | --- |
| Model | | Unstandardized Coefficients | | Standardized Coefficients | t | Sig. | 95,0% Confidence Interval for B | |
|  |  | B | Std. Error | Beta |  |  | Lower Bound | Upper Bound |
| 1 | (Constant) | 33.689 | 7.587 |  | 4.440 | <.001 | 18.698 | 48.679 |
|  | gender | -6.553 | 1.432 | -.333 | -4.577 | <.001 | -9.382 | -3.725 |
|  | age in years | -.168 | .159 | -.124 | -1.059 | .291 | -.481 | .145 |
|  | work experience in years | -.018 | .195 | -.011 | -.094 | .925 | -.404 | .367 |
|  | BMI | .834 | .182 | .326 | 4.585 | <.001 | .475 | 1.194 |
|  | subjective physical fitness day | .696 | .396 | .124 | 1.755 | .081 | -.087 | 1.479 |
| a. Dependent Variable: mean pressure depth in mm 1st + 2nd + 3rd cycle day | | | | | | | | |

**Dependent variable: mean compression depth at night**

| Variables Entered/Removed^a^ | | | |
| --- | --- | --- | --- |
| Model | Variables Entered | Variables Removed | Method |
| 1 | subjective physical fitness night, work experience in years, gender, BMI, age in years^b^ | . | Enter |
| a. Dependent Variable: mean pressure depth in mm 1st + 2nd + 3rd cycle night | | | |
| b. All requested variables entered. | | | |

| Model Summary | | | | |
| --- | --- | --- | --- | --- |
| Model | R | R Square | Adjusted R Square | Std. Error of the Estimate |
| 1 | .487^a^ | .237 | .212 | 8.5554 |
| a. Predictors: (Constant), subjective physical fitness night, work experience in years, gender, BMI, age in years | | | | |

| ANOVA^a^ | | | | | | |
| --- | --- | --- | --- | --- | --- | --- |
| Model | | Sum of Squares | df | Mean Square | F | Sig. |
| 1 | Regression | 3462.762 | 5 | 692.552 | 9.462 | <.001^b^ |
|  | Residual | 11125.599 | 152 | 73.195 |  |  |
|  | Total | 14588.361 | 157 |  |  |  |
| a. Dependent Variable: mean pressure depth in mm 1st + 2nd + 3rd cycle night | | | | | | |
| b. Predictors: (Constant), subjective physical fitness night, work experience in years, gender, BMI, age in years | | | | | | |

| Coefficients^a^ | | | | | | | | |
| --- | --- | --- | --- | --- | --- | --- | --- | --- |
| Model | | Unstandardized Coefficients | | Standardized Coefficients | t | Sig. | 95,0% Confidence Interval for B | |
|  |  | B | Std. Error | Beta |  |  | Lower Bound | Upper Bound |
| 1 | (Constant) | 41.121 | 7.726 |  | 5.323 | <.001 | 25.858 | 56.385 |
|  | gender | -5.676 | 1.467 | -.292 | -3.869 | <.001 | -8.574 | -2.778 |
|  | age in years | -.350 | .166 | -.262 | -2.114 | .036 | -.677 | -.023 |
|  | work experience in years | .192 | .203 | .117 | .945 | .346 | -.209 | .592 |
|  | BMI | .644 | .190 | .255 | 3.382 | <.001 | .268 | 1.021 |
|  | subjective physical fitness night | .874 | .469 | .135 | 1.864 | .064 | -.052 | 1.800 |
| a. Dependent Variable: mean pressure depth in mm 1st + 2nd + 3rd cycle night | | | | | | | | |
